# Selenium Supplementation in Pregnant Women with Autoimmune Thyroiditis: A Practical Approach

**DOI:** 10.3390/nu14112234

**Published:** 2022-05-27

**Authors:** Marianna Minnetti, Valentina Sada, Tiziana Feola, Elisa Giannetta, Carlotta Pozza, Daniele Gianfrilli, Andrea M. Isidori, Alessia Cozzolino

**Affiliations:** 1 Department of Experimental Medicine, Sapienza University of Rome—Policlinico Umberto I Hospital, 00161 Rome, Italy; marianna.minnetti@uniroma1.it (M.M.); valentina.sada@uniroma1.it (V.S.); tiziana.feola@uniroma1.it (T.F.); elisa.giannetta@uniroma1.it (E.G.); carlotta.pozza@uniroma1.it (C.P.); daniele.gianfrilli@uniroma1.it (D.G.); andrea.isidori@uniroma1.it (A.M.I.); 2 Neuroendocrinology, Neuromed Institute, IRCCS, 86077 Pozzilli, Italy

**Keywords:** selenium, pregnancy, thyroid, autoimmune thyroiditis

## Abstract

Selenium (Se) is an essential trace element with antioxidant and anti-inflammatory properties and a pivotal role in thyroid metabolism. Ensuring a sufficient Se supply is possible via a balanced, wholesome diet; however, Se content in foods may be different throughout geographical areas. Se supplementation is expected to improve inflammatory status in patients with autoimmune thyroiditis, especially in those with high activity, and has been demonstrated as effective in reducing the thyroid peroxidase antibodies titer. Se status seems to affect thyroid function in pregnancy, which prompts the potential role of Se supplementation in such patients. Few clinical trials have investigated the effectiveness of Se supplementation in pregnant women with thyroiditis, and their results suggest the safety and effectiveness of this element in reducing autoantibody levels and preventing postpartum thyroiditis development, although limited. Hence, more robust evidence is needed to confirm these data. The current study aims to summarize published data on the relationship between Se and thyroid status in pregnant women with thyroiditis and the potential use of Se. Moreover, an algorithm for Se supplementation is proposed for pregnant women with thyroiditis to help endocrinologists in daily clinical practice to consider Se status.

## 1. Introduction

Selenium (Se) is an essential trace element that is involved in the biosynthesis of organic compounds, namely selenomethionine and selenocysteine, being stochastically incorporated due to its nutritional availability [1,2]. Se is incorporated into proteins (named selenoproteins) and a few modified transfer RNAs. Therefore, increasing Se consumption leads to a higher Se content of proteins [3,4,5]. Twenty-five genes from the human genome encode selenoproteins. These selenoproteins have a wide range of functions, from antioxidant and anti-inflammatory activities to active thyroid hormone production [3,4].

**Figure 1:**
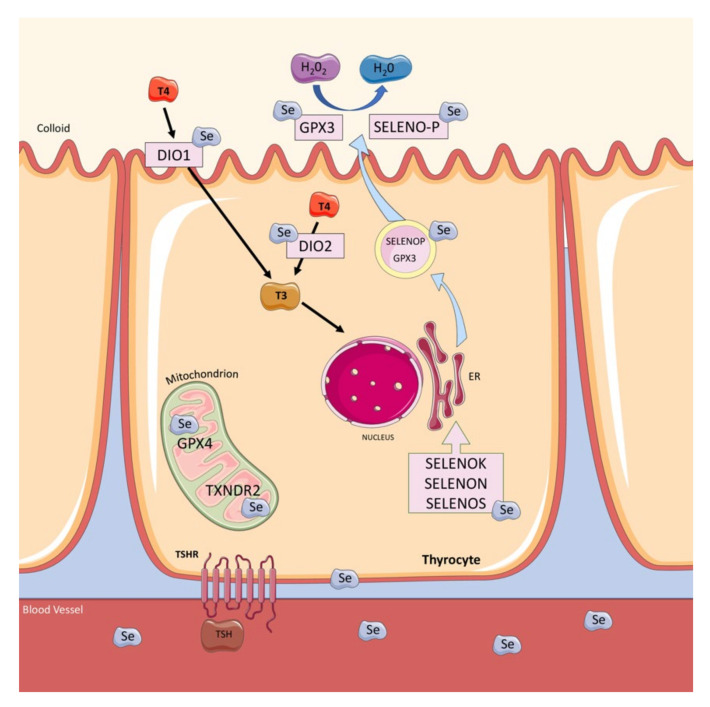
Thyroid gland selenoproteins. Selenoprotein P (SELENOP) and glutathione peroxidase 3 (GPX3) are actively secreted from the thyrocyte and exert oxidoreductase functions, protecting thyrocytes from excessive H_2_O_2_ accumulation. Other selenoproteins, including Selenoprotein K, N, and S (SELENOK, SELENON, and SELENOS, respectively) take part in the quality control pathways within the endoplasmic reticulum. Type 1 (DIO1) and 2 iodothyronine deiodinase (DIO2) regulate thyroid hormone levels, converting T4 into T3. GPX4 and thioredoxin reductase 2 (TXNRD2) are localized in the mitochondria, affording protection against the hydrogen peroxide produced by the mitochondrial respiratory chain. This figure was created using Servier Medical Art templates, which is licensed under the Creative Commons Attribution 3.0 Unported License; https://smart.servier.com (accessed on 17 February 2022).

The thyroid is the organ with the highest Se concentration of all tissue, reflecting its key role in thyroid metabolism. Thyrocytes express several selenoproteins, including the deiodinase isozymes types 1 and 2 (DIO1 and DIO2), members of the glutathione peroxidase family (GPX1, GPX3, and GPX4), the thioredoxin reductases (TXNRD1, TXNRD2, and TXNRD3), and selenoprotein (F, P, K, N, and S). These proteins are functionally active in the thyroid gland, where they exert oxidoreductase functions and regulate thyroid hormone levels [3,6] (Figure 1). Specifically, DIO1 and DIO2 convert the prohormone thyroxine (tetra-iodothyronine, T4) in the active thyroid hormone 3, 3′,5-tri-iodothyronine (T3) in thyrocytes, whereas this activation is due to DIO2 in target tissues. Deiodinase isozyme type 3 (DIO3) is not expressed in thyrocytes; however, it is expressed in the placenta [7]. Meanwhile, GPX is responsible for protecting thyrocytes from excessive H_2_O_2_ accumulation [3,4]. H_2_O_2_ is essential for the thyroid peroxidase (TPO) enzyme in the iodide oxidation process. The thyroid produces H_2_O_2_ for hormone synthesis and is exposed to free radical damage if H_2_O_2_ is not properly reduced to H_2_O by intracellular defense mechanisms or during the hormone synthesis process. Se deficiency leads to reduced GPX and DIO enzymatic activity and to a decreased H_2_O_2_ disposal with thyroid gland damage and the impairment of the thyroid hormone metabolism as a consequence [5]. Recent preclinical studies have provided molecular evidence that the correction of Se deficiency results in thyrocyte apoptosis modulation. Sodium selenite incubation in rat thyroid follicular cell lines can improve cell growth and proliferation and prevent apoptosis [8]. Additionally, Se exerts a protective action against H_2_O_2_-induced oxidative stress in human thyrocytes [9].

Oxidative stress has been advocated as a key pathogenetic event in autoimmune disorder development, such as in autoimmune thyroiditis (AIT). A sufficient Se intake is required to modulate immune cells and avoid overshooting immune response [6]. AIT pathogenesis has not yet been fully clarified; however, selenoprotein amount and activity were suggested to interfere with disease risk [10,11,12]. Se supplementation is expected to improve the inflammatory status in patients with AIT, especially in those with high activity [5]. A suggested mechanism for the potential beneficial effect of Se in AIT is linked with the role of selenoproteins in the immunoregulatory processes involving cytokine production and T cell activity [13,14].

Finally, pregnancy is a condition that strengthens demand for micronutrients. Se is transferred from the mother to the fetus during pregnancy, leading to possible Se deficiency [6]. Some selenoprotein activities such as GPXs, TXNRDs, SELENOS, and SELENOP have been implicated to reduce oxidative stress in certain pregnancy pathologies [7]. Collectively, antioxidant selenoprotein activities have been suggested as the biological rationale for Se supplementation as an effective treatment in pregnant patients with AIT.

## 2. Selenium Intake and Status

The main natural source of Se is food, and a shortlist of Se sources in the diet is depicted in Table 1 (see REF [15,16,17] for a comprehensive list).

Ensuring a sufficient Se supply is possible via a balanced wholesome diet; however, the content of Se in foods may be different throughout geographical areas due to its concentration in the soil, climatic conditions, and methods of preparing food products [3,18,19,20]. There is no reliable way of calculating individual daily Se intake outside of laboratory-based assessments because the Se content of a given food item cannot be precisely predicted due to different variables.

The most commonly used method for Se assessment is determining its levels in plasma or serum, which reflect the Se intake of the past few days [3,21,22]. The selenoproteins, GPX3 and SELENOP (Figure 1) can be used as biologically relevant Se biomarkers in the plasma because they are responsive to minor Se deficiency [16]. Both markers appear saturated at some point—GPX3 earlier than SELENOP—when Se supply is sufficiently high [23]. Se status measurement has been found to cover a wide range and is highly influenced by the ethnicity and the geographical area in which it is determined [3,19,20].

Normal plasma Se concentrations vary according to the geographical area of adult females, from approximately 86.8 μg/L (1.1 μmol/L) in Europe to 126.3 μg/L (1.6 μmol/L) in the United States of America [24]. The Food and Nutrition Board at the Institute of Medicine of the National Academies, United States, has recommended 55 μg of Se/day as a dietary allowance for 19–50-year-old females [25]. In 2015, German, Austrian, and Swiss nutrition societies revised the reference values for Se consumption to 60 μg/day for females [26]. Se levels may fall during pregnancy due to plasma volume expansion [24]. A reference range for Se in pregnant women has not yet been defined. In 2007, Alvarez found a much higher mean serum Se in the first trimester (109 μg/L), which significantly decreased at term (85.3 μg/L) in Spanish pregnant women [27]. In 2019, Grieger and coworkers reported a mean serum Se concentration of 57.6 μg/L at 15 weeks of gestation in 894 primiparous women from Australia without fertility problems [28].

Se is widely available as a supplement, especially in selenomethionine forms [25]. A major limitation in determining standard reference ranges of trace elements during pregnancy is the widespread use of supplement intake by pregnant women. A recent study by Pop and colleagues reports on the first-trimester reference range of plasma Se assessed at 12 weeks of gestation in 2041 pregnant women, including 544 women who were not taking Se-containing supplements. The reference range of the total sample was 59.2–103.4 μg/L, with women not taking Se supplements having a significantly lower mean Se concentration (75.8 μg/L) than those with supplement intake (81.3 μg/L). The study results suggest that a daily dose of 27.5–55 μg Se increases plasma Se concentrations [24].

Some people may show inadequate Se intake. This demographic comprises populations living in countries whose diet primarily consists of vegetables grown in low-Se areas (i.e., Europe, China, Russia, Middle East, and New Zealand) [26,29,30,31,32], vegetarian and vegan populations [33,34], and patients on hemodialysis [35].

Notably, both Se deficiency and excess have been associated with adverse health effects [2,36]. Low Se has been associated with inflammatory conditions, mood and cognitive disorders, cardiovascular diseases, increased viral virulence, and impaired fertility, in addition to thyroid disorders [2,3]. On the other side, selenosis (overexposure to Se) was described in China for the first time in 1983, with hair and nail loss as the most frequent symptoms, and a dosage of 400 μg has been established as the maximum safe daily dietary Se intake [37,38]. Moreover, Se supplements of 200 μg/day, which are generally considered safe, have been associated with toxic effects (alopecia, dermatitis, squamous cell carcinoma, type-2 diabetes mellitus, and high-grade prostate cancer) in North Americans [2]. Ultimately, a U-shaped relationship has been found between Se status and risk of a number of adverse conditions [2,3].

## 3. Selenium Supplementation and Autoimmune Thyroiditis

AIT is the most common human organ-specific autoimmune disease, accounting for Hashimoto’s thyroiditis in >90% of all cases. Its incidence is approximately 10% in the general population, with a male to female ratio of 1:10 [39]. Thyroglobulin antibodies (TgAb) and thyroid peroxidase antibodies (TPOAb) are the serum markers of AIT. Circulating TPOAb are present in approximately 80–90% of patients with AIT, and their positivity can be used to predict the switch from subclinical hypothyroidism to overt hypothyroidism. TgAb is detected in the serum of 60–80% of patients with AIT and determining their titer has a sensitivity of 30–50% for AIT diagnosis [40].

Se deficiency has been implicated in thyroid gland damage and impaired thyroid hormone metabolism. Moreover, based on the known Se antioxidant and anti-inflammatory properties (Figure 1), its supplementation has been promoted as a potential AIT therapy. Notably, both very low or very high Se intake (47 and 297 μg/day, respectively) alters thyroid hormone concentrations by reducing T3 in the case of low intake, and increasing T3 in the case of high Se intake [41]. Moreover, GPx activity is impaired in subjects with low–normal plasma Se concentrations because the mean concentration necessary for optimal GPx activities is approximately 90 μg/L [42]. Additionally, TPOAb reduction has been reported as dose-dependent and requires Se doses higher than 100 μg/day to maximize GPx activity [43]. Furthermore, achieving a maximal SELENOP concentration requires a plasma Se of ~120 μg/L [44].

Se supplementation has been widely explored in several trials, including patients with AIT, but with mixed results [43,45,46,47,48,49,50,51,52,53,54,55,56,57,58,59,60,61]. However, the majority of the studies found decreased TPOAb after Se treatment.

A recent systematic review and meta-analysis, including 9 randomized controlled trials and a total of 787 patients, evaluated the effectiveness of Se supplementation in AIT treatment [62]. The authors found that Se supplementation of 6- (*p* = 0.023) or 12-month (*p* < 0.001) duration significantly reduced the TPOAb titers in patients with AIT; whereas, 12-months of Se supplementation decreased the TgAb titers in these patients (*p* < 0.001). Reducing the Ab titer in pregnant patients could be particularly important, as explained in the next section. Furthermore, after treatment, mood improvement was found in the Se supplementation group compared with controls (*p* = 0.045). No serious adverse effects were recorded concerning Se supplementation, except mild gastric discomfort [62].

## 4. Selenium Status, Autoimmune Thyroiditis, and Fertility

The literature reveals scarce data on the relationship between Se status and fertility in patients with AIT and, sometimes, controversial results. Furthermore, the majority of studies have evaluated Se status or the presence of AIT in females in the early stage of pregnancy, but studies assessing the effect of Se supplementation on fertility outcomes in females with AIT are lacking. Noteworthily, unusual folliculogenesis and embryogenesis may occur in patients with elevated TPOAb levels, causing miscarriage and other fertility problems [63,64]. A lower antral follicular count was associated with low fT3 and TPOAb positivity in women with unexplained infertility or with diminished ovarian reserves [65]. However, these results have not been confirmed in other studies that compare the fertility outcomes between females with positive TPOAb and negative TPOAb [66,67].

The role of Se in male fertility [68] has been much studied; however, its function in female fertility is not yet completely understood. Se has been shown to play a role in granulosa cell proliferation through its antioxidant action [69]. Se improves GPX action, reducing the ROS production that has a pivotal role in placental oxidative stress [70,71], with proposed immunomodulatory and anti-inflammatory effects in pregnant females affected by AIT [72,73]. Hence, a study has been conducted to determine whether using immunomodulatory drugs, including the administration of a low Se dose, could improve the reproductive outcome of infertile patients with AIT. Unfortunately, the results have not yet been published (NCT03289403). However, the administration of Se in the early stages of conception in women with AIT could be hypothesized to improve their fertility, but further data will be necessary to better explain the main mechanisms underlying this process.

## 5. Autoimmune Thyroiditis in Pregnancy

Thyroid disease is a common clinical condition in pregnancy, and autoimmune thyroid disease represents the most prevalent one, with 2–17% of pregnant women presenting with positive TPOAb or TgAb [72].

Regular thyroid function during pregnancy is essential for both maternal and fetal health. The fetal thyroid gland is not functionally mature until 18–20 weeks of gestation; therefore, the fetus is strictly dependent on maternal T4 during the first trimester of pregnancy [73]. Increased levels of maternal thyroid hormone production are physiologically required during pregnancy due to estrogen-mediated thyroxine-binding globulin increases, leading to a relatively decreased active free T4 levels and the placental DIO3 deactivation of T4 to the inactive reverse T3 [74].

Untreated maternal hypothyroidism during pregnancy leads to an enhanced risk of pregnancy complications, such as an increased risk of preterm birth, low birth weight, and stillbirth [72]. Maternal hypothyroidism may also have adverse effects on the cognitive function of offspring since adequate thyroid hormone levels are crucial for neuronal migration, myelination, and other structural fetal brain modifications [75,76,77]. Maternal hypothyroidism is defined as an elevated thyroid-stimulating hormone (TSH) concentration beyond the upper limit of the pregnancy-specific reference range; when this is unavailable, a TSH upper reference limit of 4.0 mU/L may be used [72]. Females with euthyroidism and positive TPOAb before pregnancy may develop high TSH levels during pregnancy [78]; hence, not only women with preconceptional hypothyroidism, but also euthyroid patients with thyroid autoimmunity should have regular serum TSH concentration measurements during pregnancy [72]. Moreover, a two- to three-fold increase in the risk of pregnancy loss has been found in patients with positive thyroid autoantibodies, although the exact underlying mechanism still remains unclear [79,80,81,82,83]. Furthermore, an increased risk of preterm birth has been found in women with normal thyroid function and the presence of thyroid autoantibodies [82,84].

Well-established evidence and consensuses are available on the need to treat women with overt hypothyroidism during pregnancy [72]. Regarding subclinical hypothyroidism, a condition with mildly elevated TSH and peripheral thyroid hormone levels within the normal reference laboratory range, levothyroxine (LT4) therapy is recommended for (1) TPOAb-positive females with a TSH greater than the pregnancy-specific reference range, and (2) TPOAb-negative females with a TSH greater than 10.0 mU/L. Furthermore, LT4 therapy may be considered for (1) TPOAb-positive females with TSH levels of >2.5 mU/L and (2) TPOAb-negative females with TSH levels greater than the pregnancy-specific reference range and below 10.0 mU/L [72].

Moreover, euthyroid females with positive TPOAb and/or TgAb may develop postpartum thyroiditis (PPT) [85], a painless destructive thyroiditis due to the immunity rebound within the first postpartum year [85,86]. Noteworthily, patients with the highest TPOAb titers during pregnancy have the highest PPT risk [87].

In conclusion, an effective treatment to prevent them is still unavailable, although evidence has suggested that euthyroid females with AIT have a high risk of experiencing adverse obstetric outcomes. This was further confirmed by the recent T4LIFE trial, which showed that LT4 administration did not result in higher live birth rates in patients with AIT with euthyroidism with recurrent pregnancy loss [88], thus leading to research on other treatment strategies.

## 6. Selenium Status, Thyroid, and Pregnancy

Noteworthily, Se is essential for a healthy pregnancy [7] and low Se status has been associated with adverse pregnancy outcomes, such as miscarriages, neural tube defects, premature birth, low birth weight, preeclampsia, glucose intolerance, gestational diabetes, and even diaphragmatic hernia [89]. Moreover, women with Se deficiency showed an approximately eight-fold higher risk of preterm delivery and a low birth weight infant compared to females with normal Se concentrations [89].

Clinical conditions associated with declined Se status, such as pregnancy, show a considerable overlap to potential triggers for AIT development, and these processes may be causally linked. Therefore, a declined Se below a certain threshold may impair the balance from regular lymphocyte function to self-tolerance disruption, triggering autoreactive processes [6].

Several studies have evaluated the correlation between Se status and thyroid outcomes in pregnancy, with the majority of them suggesting better Se status is linked with better thyroid function [24,90,91,92,93]. Table 2 summarizes the details of studies that evaluate Se status and thyroid outcomes in pregnant women.

A cross-sectional study by Arikan evaluated Se levels in the plasma of 37 hyperthyroid (group 1) and 70 healthy (group 2) pregnant women to investigate the association between maternal plasma Se concentrations and thyroid hormone levels. Se levels were significantly lower in hyperthyroid than in healthy females (*p* < 0.05) with a positive correlation between Se and FT4 in group 1 and with TSH in group 2 (*p* < 0.05), supporting the hypothesis that hyperthyroidism is associated with decreased antioxidant response [90].

A recent prospective birth cohort study by Guo et al. evaluated Se status in 1931 pregnant women and found a non-linear association between maternal Se and TSH levels and, adjusting for potential confounders, when maternal serum Se levels were <103.7 μg/L, each unit increase in Se levels (μg/L) was significantly associated with a decrease of 0.014 μIU/mL in TSH levels (*p* = 0.023). Moreover, maternal TSH levels were significantly inversely associated with infant birth weight (*p* = 0.010). Therefore, the authors speculated that low Se status during pregnancy may be associated with low thyroid function related to low birth weights [91].

Hofstee et al. investigated the relationship between maternal Se and thyroid hormone status during pregnancy by utilizing data from a retrospective, cross-sectional study and dividing 63 women into three groups as follows: 21 with the lowest serum Se concentrations (51.2 ± 1.2 μg/L), 21 with mean concentrations that represented the entire cohort (78.8 ± 0.4 μg/L), and 21 with optimal serum Se concentrations (106.9 ± 2.3 μg/L). Iodine concentrations between the mean and the low serum Se groups were not different, whereas women in the optimal serum Se group had significantly higher iodine concentrations (*p* = 0.016). Women with low serum Se concentrations demonstrated reduced FT3 levels (*p* < 0.05) and increased TPOAb (*p* < 0.01), and serum Se was positively correlated with FT3 (*p* < 0.05) and negatively correlated with TPOAb (*p* < 0.001). Furthermore, the FT4/TSH ratio was increased in the low-Se cohort. Pregnancy disorder incidence, most notably gestational diabetes mellitus, was increased within the low-Se cohort [93].

Pop et al. reported on the first-trimester reference ranges of plasma mineral Se/zinc/copper concentrations concerning FT4, TSH, and TPOAb, assessed at 12 weeks of gestation in 2041 pregnant women, including 544 women not taking mineral supplements. Plasma Se concentrations negatively correlated with logFT4 (*p* = 0·014) and positively correlated with logTSH (*p* = 0·001), and women taking mineral supplements were 1.46 times less likely to have elevated TPOAb at 12 weeks of gestation [24].

Conversely, Ambroziak et al. assessed Se status in the form of serum Se and SELENOP concentrations in 74 consecutively recruited pregnant women—45 healthy, and 29 with autoimmune thyroid disease—and concluded that a high proportion of women developed a severe Se deficit during pregnancy, irrespective of autoimmune thyroid disease, in a geographic area of habitually low Se intake (i.e., Poland). Furthermore, an increased iodine excretion between the first and third trimester was observed in the group of women with AIT (*p* < 0.01), probably due to the co-excretion of iodine derived from the multivitamin tablets and LT4 given to 48% of women in this group [92].

**Figure 2:**
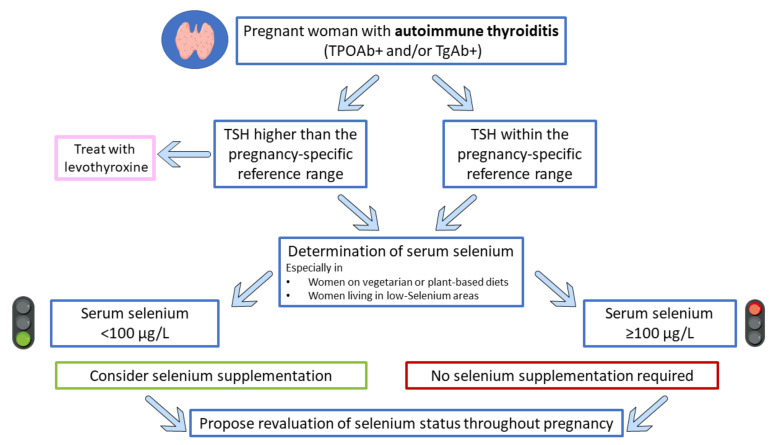
Proposed algorithm for selenium supplementation in pregnant women with thyroiditis. This figure was created using Servier Medical Art templates, which is licensed under the Creative Commons Attribution 3.0 Unported License; https://smart.servier.com (accessed on 17 February 2022).

Definitively, Se status seems to affect thyroid function in pregnancy, suggesting a potential role of Se supplementation in this setting of patients. Notably, the 2017 American Thyroid (ATA) association guidelines recommend against Se supplementation in pregnant patients with AIT (a weak recommendation with moderate-quality evidence), although both the presence of AIT and low Se status can lead to adverse obstetric outcomes as reported before, and recent evidence suggests promising results of Se supplementation [72,89,94]. Additionally, to avoid excessive Se intake, which is possibly correlated with several conditions [36], the measurement of Se status could be beneficial before supplementation to achieve an optimum Se status and reduce disease risk (Figure 2).

## 7. Selenium Supplementation in Pregnant Women with Autoimmune Thyroiditis: Has the Time Come?

To date, three placebo-controlled trials have evaluated the impact of Se supplementation on thyroid function in pregnancy. Table 3 summarizes the details of the Se supplementation trials evaluating thyroid outcomes in pregnant women.

Negro et al. examined the influence of Se supplementation both during and after pregnancy in terms of thyroidal autoimmune pattern and function. This prospective, randomized, placebo-controlled study enrolled 77 TPOAb-positive women receiving selenomethionine 200 μg/day (group S1), 74 TPOAb-negative women receiving a placebo (group S0), and 81 TPOAb-negative age-matched women as the control group (group C). All the participants were advised to use iodized salt at the start of the study. At the thyroid ultrasound evaluation, the parenchymal echogenicity was classified as normal (grade 0) or mild thyroiditis (grade 1), moderate thyroiditis (grade 2), or advanced thyroiditis (grade 3). After delivery, patients were considered to have hypothyroidism if their TSH values were above the normal range, whether or not their FT4 values were below or within the normal range. The authors found that PPT (*p* < 0.01) and permanent hypothyroidism (*p* < 0.01) were significantly lower in group S1 compared with S0. No adverse effects due to excess Se intake were observed in the Se-treated group [95].

These data have been partially confirmed by a more recent Italian study by Mantovani et al., the SERENA study, a multicenter, randomized, double-blind, placebo-controlled trial that included 45 pregnant women with thyroiditis who were randomly assigned to selenomethionine 83 µg/day or placebo. The authors found a significant autoantibody reduction in the treatment group (*p* < 0.01) and a rebound in their titer in the placebo group after delivery (*p* < 0.01). No differences were found in adverse pregnancy outcomes [97].

Conversely, Mao et al. evaluated the effect of a Se nutritional dose (60 μg/day) on thyroid autoimmunity and thyroid function in pregnant women with mild-to-moderate iodine deficiency in the United Kingdom and found that Se supplementation was no more beneficial than placebo in reducing TPOAb levels, and that it determined a decrease in both TSH and FT4 levels in women with thyroiditis. Meanwhile, the authors found that Se supplementation improved thyroid function in women with thyroiditis, for which the decreased TSH levels became almost significantly lower than in females with thyroiditis on the placebo (*p* = 0.05). Moreover, they found that the iodine-to-creatinine ratio in the Se group was significantly lower than that in the placebo group (*p* = 0.034), but only in women without thyroiditis [96].

The different findings between the study by Mao et al. and the other two aforementioned studies have many reasonable explanations as follows: (1) the number of TPOAb-positive women was lower in the United Kingdom study compared to the Italian ones (25 vs. 151 and 25 vs. 45), resulting in a lower chance of seeing an effect; (2) baseline TPOAb levels were much lower in the study by Mao compared to the other (110 vs. 627 U/mL and 110 vs. 352 U/mL) and a higher concentration is needed for better treatment responses; (3) the Se dose was lower (60 vs. 200 μg/day and 60 vs. 83 μg/day) in Mao’s study and was probably too low to sustain an effect; (4) none of the study participants received LT4 treatment in Mao’s study in contrast to 20% of the TPOAb-positive women in Negro’s study, and 71% of the women in Mantovani’s study had a potential LT4 additive effect in reducing TPOAb levels in patients with thyroiditis. 

The results derived from these trials are not completely consistent; however, they suggest that Se supplementation in pregnant women with positive TPOAb is safe and could effectively reduce autoantibody levels and prevent PPT development, a clinical condition with a prevalence ranging from 33% to 50% [98]. The higher the autoantibody level, the higher the risk of developing PPT and, consequently, hypothyroidism that occurs from 3 to 12 months after delivery [87]. More robust evidence is needed to confirm these data.

Finally, a recent Italian study by Negro et al. evaluated the use of Se in daily clinical practice among Italian endocrinologists by a web-based survey that investigated the use of Se supplementation in different clinical conditions. The survey results showed that approximately 40% of participants had suggested Se use for patients with AIT who were planning a pregnancy or who were already pregnant, with different aims as follows: 39.9% to prevent hypothyroidism development; 24.7% to prevent PPT; 30.7% to reduce TPOAb titers. Moreover, almost 30% of the respondents suggested a Se supplementation of <100 μg/day, 60% recommended 100–200 μg/day, and 10% suggested >200 μg/day [99]. The aforementioned survey results suggest that Se use is widely considered in daily clinical practice, at least in Italy, beyond what is supported by evidence-based medicine and patients’ Se status.

In conclusion, it is important to underline that Se supplementation is not recommended by the 2017 ATA guidelines for the treatment of TPOAb-positive women during pregnancy. The authors explained that any generalized recommendation was unreliable due to conflicting data regarding Se supplementation published before 2017 [72]. Therefore, due to adverse health effects related to both Se deficiency and excess [2,36], special care around Se supplementation during pregnancy is needed, and Se status assessment before Se administration could be an effective and safe option to prevent impaired thyroid function during pregnancy and in the postpartum period [72,89]. In this contest, additional studies are needed to clarify how endocrinologists can appropriately determine Se treatment for AIT management in pregnancy. Figure 2 shows an algorithm proposal indicating a good practical approach for considering Se status in these cases.

## 8. Conclusions

Se is an essential trace element with a pivotal role in thyroid metabolism. The antioxidant activities of selenoproteins have been suggested as the biological rationale for the use of Se supplementation as an effective treatment in these patients, since oxidative stress has been advocated as a key pathogenetic event in AIT development. Se supplementation has been demonstrated as being effective in reducing the TPOAb titer. Moreover, Se status seems to affect thyroid function during pregnancy, prompting investigation into the potential role of Se supplementation in this setting. To date, few clinical trials have investigated the effectiveness of Se supplementation in pregnant women with thyroiditis and their results suggest its safety and effectiveness in reducing autoantibody levels and preventing the development of postpartum thyroiditis, although these studies are limited. However, Se exhibits U-shaped effects, which should be considered when Se supplementation is planned. Therefore, an individualized approach taking Se status into account may be a good strategy to improve thyroid function in women with AIT both during pregnancy and in the postpartum period.

## Figures and Tables

**Table 1:** Examples of selenium food sources.

Food	Se Content (µg/g Fresh Weight)
Brazil nuts	~0.85–7
Tuna (in oil)	~0.8
Chicken	~0.6
Sardines	~0.6
Lamb	~0.3-0.4
Shellfish	~0.4–1.3
Beef	~0.35–0.5
Salmon	~0.2–0.4
Ham	~0.2
Eggs	~0.2
Milk products	~0.1–0.5

**Table 2:** Details of studies evaluating selenium status and thyroid outcomes in pregnant women.

First Author, Year	Country	Study Design	Study Population	No. of Patients	Gestational Age	Outcomes	Main Results
Arikan et al. 2015 [90]	Turkey	Cross-sectional study	Healthy pregnant vs. hyperthyroid pregnant	107: 70 healthy (group 1); 37 hyperthyroid (group 2)	First trimester	TSH, FT3, FT4, serum Se	Significantly higher FT3 and FT4 levels and significantly lower TSH and Se levels in group 2 than in group 1; positive correlation between Se and FT4 in group 1 and with TSH in group 2
Ambroziak et al. 2017 [92]	Poland	Prospective study	Healthy pregnant and pregnant women with AITD	74: 45 healthy; 39 AITD	First, second and third trimester	TSH, FT3, FT4, TPOAb, TgAb, thyroid US, serum Se and SELENOP	Relatively low serum Se and SELENOP levels in both healthy and AITD; from first to third trimester TPOAb and TgAb declined in AITD, but this was unrelated to Se status
Guo et al. 2021 [91]	China	Prospective cohort study	Pregnant	1931	28–36 wks	TSH, serum Se, birth weight and length	Serum Se levels <103.7 μg/L, each unit increase significantly associated with a decrease of 0.014 μIU/mL in TSH; maternal TSH levels inversely associated with infant birth weights
Hofstee et al. 2021 [93]	Australia	Retrospective cross-sectional study	Pregnant euthyroid	63: 21 with low Se; 21 with mean Se; 21 with optimal Se	26–30 wks	TSH, FT3, FT4, TPOAb, serum Se	Females with low Se concentrations showed reduced FT3 and increased TPOAb and incidence of pregnancy disorders; Se levels positively correlated with FT3 and negatively correlated with TPOAb
Pop et al. 2021 [24]	The Netherlands	Longitudinal prospective study	Pregnant	2041: 1479 taking mineral supplements; 544 not taking mineral supplements	12 wks	TSH, FT4, TPOAb, serum Se, Zn and Cu	Negative correlation between Se levels and logFT4; positive correlation between Se levels and logTSH; women taking supplements were 1.46 times less likely to have elevated TPOAb at 12 wks

Se—selenium; wks—weeks; AITD—autoimmune thyroid disease; SELENOP—selenoprotein P; US—ultrasound.

**Table 3:** Details of selenium supplementation trials evaluating thyroid outcomes in pregnant women.

First Author, Year	Country	No. of Patients (Selenium/Placebo/Control)	Selenium Supplementation	Duration of Treatment (Months)	Patients Requiring LT4 during Pregnancy (%)	Outcomes	Main Results
Negro et al. 2007 [95]	Italy	232 (77/74/81)	selenomethionine 200 μg/day	18	33 (14.2)	TSH, FT4, TPOAb, thyroid US, PPT	Lower prevalence of PPT and permanent hypothyroidism
Mao et al. 2016 [96]	UK	230 (120/110)	selenium 60 μg/day	6	None	TSH, FT4, TPOAb, TgAb	Decrease in TSH and FT4 during pregnancy but no effect on TPOAb
Mantovani et al. 2019 [97]	Italy	45 (21/24)	selenomethionine 83 μg/day	12	13 * (28.9)	TSH, FT3, FT4, TPOAb, TgAb, thyroid US, HRQoL	Decrease in TPOAb and TgAb in the Se group but increase in the PLB group at PP

* Ten patients required LT4 dosage adjustment and three patients began LT4 treatment; PPT—postpartum thyroiditis; PLB—placebo; PP—postpartum.

## Data Availability

Not applicable.
